# Using the Behavioral Risk Factor Surveillance System to Assess Mental Health, Travis County, Texas, 2011–2016

**DOI:** 10.5888/pcd16.180449

**Published:** 2019-03-14

**Authors:** Haruna Miyakado-Steger, Sarah Seidel

**Affiliations:** 1 Austin Public Health, Austin, Texas

## Abstract

The purpose of this study was to explore using BRFSS data to assess mental health in a community. We describe and compare adults reporting diagnosed depression and adults reporting poor mental health and the associations of chronic diseases with each condition in Travis County, Texas. Significant associations between each mental health condition and chronic diseases existed; however, demographics, risk behaviors, and health care access differed between those reporting depression and those reporting poor mental health. Assessing poor mental health in addition to diagnosed depression can identify at-risk and potentially undiagnosed populations.

**Summary**

**What is already known on this topic?**

Mood disorders, of which the most common is depression, are prevalent among US adults and can exacerbate many chronic health conditions.

**What is added by this report?**

We explored Behavioral Risk Factor Surveillance System (BRFSS) data as a tool for assessing mental health in a community. By assessing poor mental health in addition to diagnosed depression, we identified at-risk and potentially undiagnosed populations.

**What are the implications for public health practice?**

The finding that individuals reporting chronic conditions had a higher odds of reporting depression and poor mental health indicates that interventions must address chronic disease and poor mental health or diagnosed mental health conditions such as depression in tandem.

## Objective

An estimated 1 in 10 adults have some type of mood disorder, the most common being depression (1). Additionally, both mood disorder and depression can exacerbate many chronic health conditions (1–5). Therefore, identifying populations at risk for mental health conditions is important for prevention and management of chronic diseases. The objective of this study was to explore using Behavioral Risk Factor Surveillance System (BRFSS) data as a tool for assessing mental health in a community. We describe and compare adults who reported diagnosed depression and adults reporting poor mental health and the associations of chronic diseases with each condition in Travis County, Texas.

## Methods

BRFSS is a federally supported landline and cellular telephone survey (6) and its data are often the only ones available to measure prevalence of chronic conditions and risk factors at a local population level. We used BRFSS data for Travis County residents from 2011 through 2016 to assess mental health status. BRFSS has 2 questions to assess mental health. Depression is measured by a yes/no response to the question “Has a doctor, nurse, or other health professional ever told you that you have a depressive disorder including depression, major depression, dysthymia, or minor depression?” Poor mental health is measured by a “none to less than 14 days” or “14 or more days” response to the question, “Now thinking about your mental health, which includes stress, depression, and problems with emotions, for how many days during the past 30 days was your mental health not good?” Demographic characteristics included race/ethnicity, sex, age, education, and income. Health care access was measured by whether respondents had a personal doctor. Self-reported risk factors included heavy drinking and smoking status.

Five chronic diseases were individually assessed for their association with each mental health condition. Cardiovascular disease, diabetes, obesity, and chronic obstructive pulmonary disease (COPD) were measured by individuals reporting that they had been told by a health care professional they have the disease. Asthma was defined as having been told by a health care professional that they had asthma and reporting that they currently have asthma.

We stratified prevalence of depression and poor mental health by demographic characteristics, health care access, risk behaviors, and chronic disease. We used logistic regression models to determine associations between each chronic disease and depression or poor mental health, respectively, and adjusted for demographics (sex, age group, and race/ethnicity), having a personal doctor, smoking status, and heavy drinking. Significance for bivariate analysis was set at *P* < .05, and significance for multivariate analysis was set at *P* < .01.

## Results

More adults reported diagnosed depression (16%) than poor mental health (10%) (Table 1). White respondents had the highest prevalence of diagnosed depression, and black respondents had the highest prevalence of poor mental health. Adults aged 45 to 64 reported the highest prevalence of diagnosed depression, and adults aged 18–44 reported the highest prevalence of poor mental health.

**Table 1:** Prevalence of Depression and Poor Mental Health by Demographic Characteristics, Health Care Access, Risk Behaviors, and Chronic Health Conditions, Adults in Travis, County, Texas, BRFSS 2011–2016

Category	Depression (N = 131,540)	Poor Mental Health (N = 79,652)
	% (95% Confidence Interval)	
**Total population**	16.4 (15.0–17.8)	10.0 (8.7–11.3)
**Demographic Characteristics**		
**Race/ethnicity**		
White	20.4^a^ (18.4–22.4)	9.1 (7.7–10.4)
Black	16.0 (11.5–20.5)	15.5 (9.8–21.3)
Hispanic	9.8^a^ (7.5–12.0)	8.8 (6.3–11.3)
**Sex**		
Female	20.7^a^ (18.5–22.9)	11.8 (9.9–13.8)
Male	12.1^a^ (10.2–13.9)	8.2 (6.6–9.9)
**Age, y**		
18–44	15.9 (13.6–18.1)	11.1^a^ (9.1–13.1)
45–64	18.7^a^ (16.5–20.8)	9.1 (7.5–10.8)
≥65	13.3^a^ (11.3–15.4)	6.9^a^ (5.0–8.8)
**Education**		
Less than high school graduate	12.6 (9.0–16.3)	10.9 (6.7–15.0)
High school graduate	16.3 (12.6–19.9)	14.1^a^ (10.4–17.9)
Some college	17.2 (14.2–20.2)	11.3 (8.7–13.9)
College graduate	17.1 (15.2–19.0)	6.4^a^ (5.2–7.7)
**Annual income, $**		
<25,000	21.0^a^ (17.6–24.5)	16.3^a^ (12.8–19.8)
25,000 to <75,000	16.8 (14.2–19.4)	9.5^a^ (7.5–11.5)
≥75,000	14.3^a^ (12.0–16.5)	5.8^a^ (4.3–7.4)
**Health Care Access**		
**Having a personal doctor**		
Yes	17.1 (15.5–22.3)	9.2 (7.7–10.6)
No	14.7 (11.9–17.5)	11.7 (9.1–14.4)
**Risk Behavior**		
**Heavy drinking^c^**		
Yes	25.4^a^ (19.0–31.9)	13.0 (7.6–18.3)
No	16.0^a^ (14.4–17.5)	9.3 (8.0–10.7)
**Smoking status**		
Current	27.6^a^ (22.4–32.8)	19.4^a^^,^ b (14.8–24.0)
Former	21.9 (18.7–25.1)	8.8^a^ (6.6–11.0)
Never	12.3^a^ (10.7–14.0)	8.6^b^ (7.0–10.2)
**Chronic Health Conditions^d^**		
**Cardiovascular disease**		
Yes	29.5^a^ (23.3–35.8)	19.2^a^ (12.3–26.1)
No	15.6^a^ (14.1–17.1)	9.5^a^ (8.2–10.8)
**Diabetes**		
Yes	24.3^a^ (19.3–29.3)	13.1 (9.1–17.1)
No	15.7^a^ (14.2–17.2)	9.8 (8.4–11.2)
**Obesity**		
Yes	19.4 (16.5–22.3)	13.2^a^ (10.5–16.0)
No	16.0 (14.2–17.7)	8.7^a^ (7.2–10.1)
**COPD**		
Yes	41.5^a^ (32.1–50.9)	31.4^a^ (21.1–41.7)
No	15.7^a^ (14.1–17.3)	8.7^a^ (7.3–10.0)
**Asthma**		
Yes	27.0^a^ (20.3–33.8)	21.0^a^ (14.0–28.1)
No	15.4^a^ (14.0–16.8)	9.0^a^ (7.8–10.3)

Abbreviations: BRFSS, Behavioral Risk Factor Surveillance System; COPD, chronic obstructive pulmonary disease.

a,b Nonoverlapping 95% confidence intervals were considered significant between groups within each category.

c Heavy drinking defined ≥15 drinks per week for men or ≥8 drinks per week for women.

d Cardiovascular disease includes coronary heart disease, angina, congestive heart failure, high blood pressure, and stroke; COPD includes chronic obstructive pulmonary disease, emphysema, or chronic bronchitis.

Respondents with a personal doctor had a higher prevalence of diagnosed depression than those without a personal doctor, and respondents without a personal doctor had a higher prevalence of poor mental health than respondents with one. In some cases, the population that reported depression and the population that reported poor mental health were similar (Table 1). Female respondents had a prevalence of diagnosed depression almost twice as high as male respondents and a prevalence of poor mental health that was nearly 1.5 times that of male respondents. Respondents with an annual income of less than $25,000 reported the highest prevalence of both depression and poor mental health compared with other income groups. Respondents who reported heavy drinking and current smoking had the highest prevalence of both diagnosed depression and poor mental health. Prevalence of depression and poor mental health were significantly higher among respondents with chronic health conditions than among individuals without these conditions (Table 1). 

After controlling for demographic characteristics, risk behaviors, and having a personal doctor, the odds of reporting depression or the odds of reporting poor mental health were significantly higher for individuals reporting cardiovascular disease, diabetes, COPD, and asthma (Table 2).

**Table 2:** Adjusted Odds of Reporting Depression and Poor Mental Health Among Adults With Each Chronic Condition, Travis County, Texas, BRFSS 2011–2016

Chronic Condition	Depression		Poor Mental Health	
	Adjusted OR^a^ (95% CI)	*P* Value	Adjusted OR^a^ (95% CI)	*P* Value
**Cardiovascular disease^b^**				
Yes	2.3 (1.6–3.4)	<.001	2.9 (1.6–5.1)	<.001
No	1 [Reference]		1 [Reference]	
**Diabetes**				
Yes	2.1 (1.6–3.0)	<.001	1.8 (1.2–2.7)	.003
No	1 [Reference]		1 [Reference]	
**Obesity**				
Yes	1.3 (1.0–1.7)	.02	1.7 (1.2–2.4)	<.001
No	1 [Reference]		1 [Reference]	
**COPD^b^**				
Yes	2.7 (1.7–4.2)	<.001	4.0 (2.3 –7.1)	<.001
No	1 [Reference]		1 [Reference]	
**Asthma**				
Yes	1.7 (1.1–2.6)	.015	2.4 (1.5–4.0)	<.001
No	1 [Reference]		1 [Reference]	

Abbreviations: BRFSS, Behavioral Risk Factor Surveillance System; CI, confidence interval; COPD, chronic obstructive pulmonary disease; OR, odds ratio.

a ORs adjusted for demographics (sex, age group, race/ethnicity), having a personal doctor, smoking status, and heavy drinking. Significance set at *P* < .01.

b Cardiovascular disease includes coronary heart disease, angina, congestive heart failure, high blood pressure, and stroke; COPD includes chronic obstructive pulmonary disease, emphysema, or chronic bronchitis.

## Discussion

To our knowledge, this study is the first to describe using BRFSS data on diagnosed depression and poor mental health for a more complete assessment of mental health in a population. Although the terms are often used interchangeably, poor mental health and mental illness are not the same thing (1). However, poor mental health is a risk factor for undiagnosed mental health conditions, including depression, which is the most commonly diagnosed mental health condition. Temporary feelings of sadness and other symptoms that last longer than 2 weeks may be diagnosed as depression (7); therefore, more than 14 days of poor mental health in the past 30 days could indicate an undiagnosed mental health condition. Assessing poor mental health in addition to diagnosed depression can assist public health practitioners in determining populations who may be at risk for undiagnosed, untreated, or unmanaged mental health conditions.

Our study has limitations. Findings cannot be generalized to other locations; however, our methods offer practitioners a useful tool for assessing mental health and its associations with chronic disease in their areas. BRFSS data are self-reported and do not represent individuals without a telephone or mailing address; they are also subject to social desirability bias, especially as it relates to stigmatized conditions. Therefore, poor mental health and diagnosed depression may be underreported.

Our findings suggest that programs focusing on mental health in Travis County should ensure that they include black adults, younger adults, adults with lower education levels, and adults without a personal doctor. Access to care should be considered in assessing mental health in a population, because individuals without a personal doctor may not receive diagnosis or subsequent treatment. Additionally, the finding that individuals reporting chronic conditions had higher odds of reporting depression and poor mental health even after controlling for covariates emphasizes that interventions must address chronic disease and poor mental health or diagnosed mental health conditions such as depression in tandem.
